# Transcriptional Profiling of Disease-Induced Host Responses in Bovine Tuberculosis and the Identification of Potential Diagnostic Biomarkers

**DOI:** 10.1371/journal.pone.0030626

**Published:** 2012-02-16

**Authors:** Elihu Aranday-Cortes, Philip J. Hogarth, Daryan A. Kaveh, Adam O. Whelan, Bernardo Villarreal-Ramos, Ajit Lalvani, H. Martin Vordermeier

**Affiliations:** 1 TB Research Group, Animal Health & Veterinary Laboratories Agency Weybridge, New Haw, Addlestone, Surrey, United Kingdom; 2 Tuberculosis Immunology Group, National Heart and Lung Institute, Imperial College London, London, United Kingdom; Fundació Institut d'Investigació en Ciències de la Salut Germans Trias i Pujol. Universitat Autònoma de Barcelona. CIBERES, Spain

## Abstract

Bovine tuberculosis (bTb) remains a major and economically important disease of livestock. Improved ante-mortem diagnostic tools would help to underpin novel control strategies. The definition of biomarkers correlating with disease progression could have impact on the rational design of novel diagnostic approaches for bTb. We have used a murine bTb model to identify promising candidates in the host transcriptome post-infection. RNA from *in vitro*-stimulated splenocytes and lung cells from BALB/c mice infected aerogenically with *Mycobacterium bovis* were probed with high-density microarrays to identify possible biomarkers of disease. In antigen-stimulated splenocytes we found statistically significant differential regulation of 1109 genes early (3 days) after infection and 1134 at a later time-point post-infection (14 days). 618 of these genes were modulated at both time points. In lung cells, 282 genes were significantly modulated post-infection. Amongst the most strongly up-regulated genes were: granzyme A, granzyme B, cxcl9, interleukin-22, and ccr6. The expression of 14 out of the most up-regulated genes identified in the murine studies was evaluated using *in vitro* with antigen-stimulated PBMC from uninfected and naturally infected cattle. We show that the expression of cxcl9, cxcl10, granzyme A and interleukin-22 was significantly increased in PBMC from infected cattle compared to naïve animals following PPD stimulation *in vitro*. Thus, murine transcriptome analysis can be used to predict immunological responses in cattle allowing the prioritisation of CXCLl9, CXCL10, Granzyme A and IL-22 as potential additional readout systems for the ante-mortem diagnosis of bovine tuberculosis.

## Introduction

Bovine tuberculosis (bTb), mainly caused by mainly by *Mycobacterium bovis*, remains an economically important disease of livestock such as cattle [1] and is also a disease of zoonotic importance. Host biomarkers for bTb are needed urgently in several areas to underpin disease control strategies. For example, correlates of disease and/or pathology could improve the sensitivity of immunological ante-mortem diagnosis which is at present mainly based on tuberculin skin testing and ancillary blood tests. Furthermore, predictors of protection and correlates of protective immunity after vaccination would greatly facilitate vaccine development.

Although IFN-γ production has been a useful tool for the blood-based detection of *M. bovis* infection in cattle and other species [2], [3], [4] as well as for the detection of *M. tuberculosis* infected humans, additional biomarkers could improve the accuracy of *in vitro* blood tests [5]. For example, it has been shown recently that simultaneous measurement of antigen-stimulated IL-1β and TNF-α production enhances IFN-γ test sensitivity to diagnose bTb in cattle [6]. Previously, we have shown in a mouse model of *M. bovis* infection that studying cellular immune responses in BCG vaccinated compared to control animals can guide the study of corresponding responses found in cattle [7]. Therefore, in the present study we applied a systematic approach to discover potential diagnostic biomarkers based on the definition of biomarkers in a cost-effective murine bTb model followed by validation of promising markers in cattle.

The paucity of reagents for cattle for the study of immunologically relevant markers by antibody-based assays such as the luminex multiplex system applied to human tuberculosis (e.g. [8]), makes host transcriptome analysis in cattle an attractive alternative. Therefore, in this study we report our application of microarray technology in combination with murine *M. bovis* infection experiments to select the most strongly up-regulated genes expressed from the whole transcriptomes of lung and spleen cells to predict biomarkers of disease in *M. bovis* infected cattle.

## Results

### Gene expression profiling of early disease in spleen and lung from mice infected with *M. bovis*

**Figure 1:**
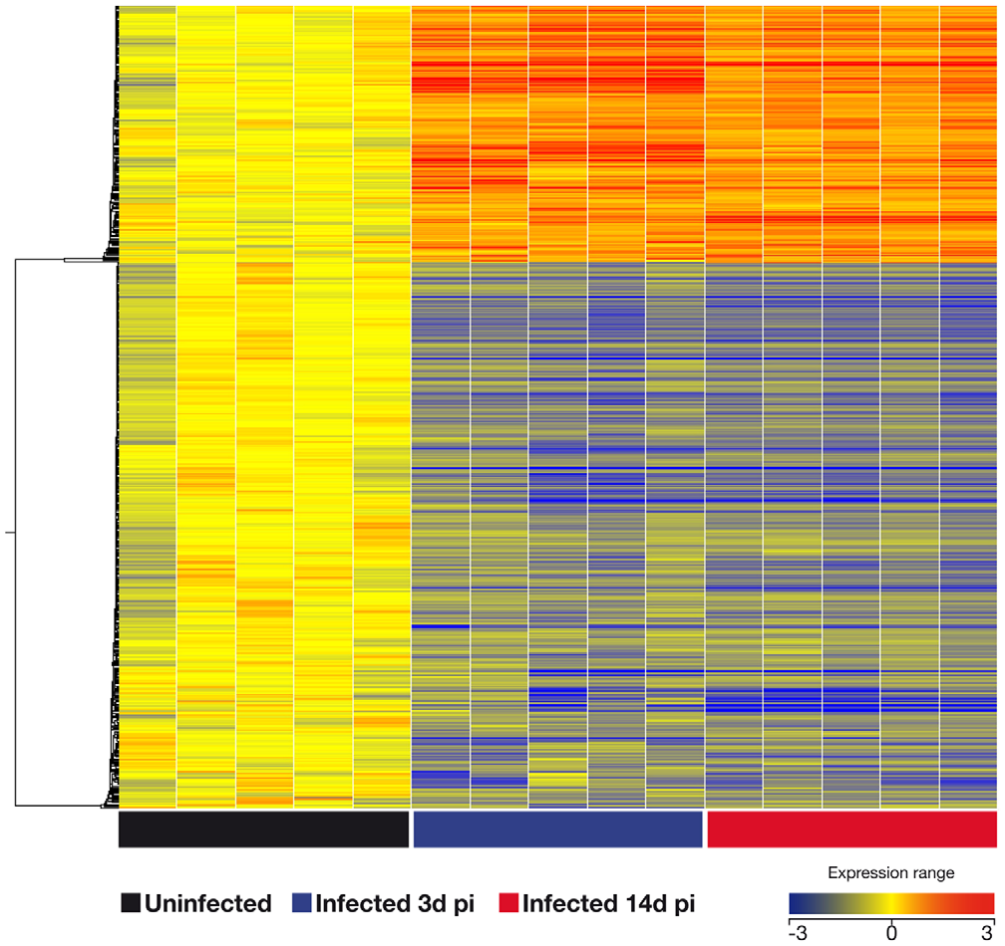
Spleen gene signature after 3 and 14 days after infection with *M. bovis*. The global transcriptional response in spleen cells of mice infected with *M. bovis* was compared to responses in uninfected mice. Genes were considered significantly modulated when their corrected p-values were below 0.05 with more than 2-fold change of expression at both time points. After 3 and 14 days post-infection (blue square and red squares, respectively at the bottom of graph), the mice were euthanized and their splenocytes were stimulated in vitro for 3 days with M7 protein pool (*see* *Materials and Methods*). Black squares: Naïve control mice. Unsupervised hierarchical cluster was performed using a centroid linkage with a Person centered measure showing that 618 of these genes were modulated at both time points (see table S1 for list of these genes, with genes significantly modulated at both time points highlighted in bold).

In order to identify potential biomarkers of tuberculosis infection, two groups of 5 BALB/C mice each were infected with *M. bovis*. After 3 and 14 days post-infection (p.i.) mice were euthanized and their splenocytes stimulated *in vitro* for 3 days with a protein pool of seven defined mycobacterial antigens, termed M7. Lung cells were collected and stimulated only at the 14 day p.i. time point. Following stimulation the fold change of gene expression was established using Whole Mouse Genome Oligo Microarrays. First, we compared the global transcriptional response in the spleen of mice infected with *M. bovis* against uninfected mice, and genes were considered significant when their corrected p-values were below 0.05 with more than a 2-fold change of expression. In antigen-stimulated splenocytes we found significant modulation of 1109 genes early after infection (day 3 p.i., Table S1) and 1134 at later time-point post-infection (day 14 p.i.) (Table S1). Unsupervised hierarchical cluster was performed using a centered linkage with a Person centered measure showing that 618 of these genes were modulated at both time points (Figure 1). Amongst the genes most strongly up-regulated at both time points p.i. was granzyme A (gzmA) with 21-fold and 26-fold changes in expression in infected animals compared to naïve controls after 3 days and 14 days p.i. respectively (Table 1). Amongst the genes significantly modulated only at 14 days p..i. were histocompatibility 28 (H28), and ubiquitin D, suggesting that they are associated with early disease progression (data not shown).

**Table 1:** Expression of the most up-regulated genes found in the murine model in cattle using PBMC from uninfected (bTb-free, n = 9) and naturally with *M. bovis* infected cows (bTb, n = 11).

		Mouse data	Bovine PBMC data		
Gene Name	Gene		bTb Free	bTb	ρ-value
	Symbol	Lung	Mean ± SEM of Log(FC)	Mean ± SEM of Log(FC)	
		14 days p.i.			
Interferon gamma	IFN-γ	82.54	0.068±0.30	2.122±0.19	<0.0001*
Interlukin-22	IL-22	74.15	0.125±0.32	2.215±0.20	0.0002*
Chemokine (C-X- motif) ligand 9	Cxcl9	23.8	0.149±0.24	2.492±0.26	<0.0001*
Interleukin-17A^a^	IL-17A	NA	0.509±0.33	1.73±0.13	0.0052
Chemokine (C-X- motif) ligand 10	Cxcl10	22.13	0.079±0.19	1.626±0.28	0.0004*
Lymphocyte-activation gene 3	Lag3	12.11	0.436±0.31	0.228±0.18	0.582
Signal transducer and activator of transcription 1	Stat1	3.15	0.234±0.11	0.466±0.17	0.3746
Granzyme B	GzmB	5.84	−0.167±0.19	−0.242±0.09	0.7249
Interferon regulatory factor	Irf4	4.25	0.275±0.21	0.200±0.13	0.7664
Interferon gamma inducible protein 47	Ifi47	4.14	0.008±0.02	0.089±0.17	0.7964
Interleukin-17 receptor E receptor	IL-17RE	3.99	0.567±0.36	−0.285±0.32	0.1638

		Spleen				
		3 days p.i.	14 days p.i.			
Granzyme A	GzmA	21.27	26.58	−0.232±0.35	1.245±0.23	0.0029*
Acetylgalactosamin transferase 3	Galnt3	19.21	13.34	−0.167±0.19	−0.242±0.09	0.7249
Adenosine deaminase	Ada	18.42	12.09	−0.641±0.12	−0.669±0.26	0.9366
Killer cell lectin-like receptor subfamily K member 1	Klrk1	5.02	4.81	1.206±0.59	−0.058±0.39	0.1141

The data are represented as mean (± SEM) fold changes in expression after stimulation of PBMC with bovine PPD-B. Significance level for comparison of results in bTb-free and infected animals: p -value≤0.0033 (*).

a Used as positive control.

NA, not applicable.

**Figure 2:**
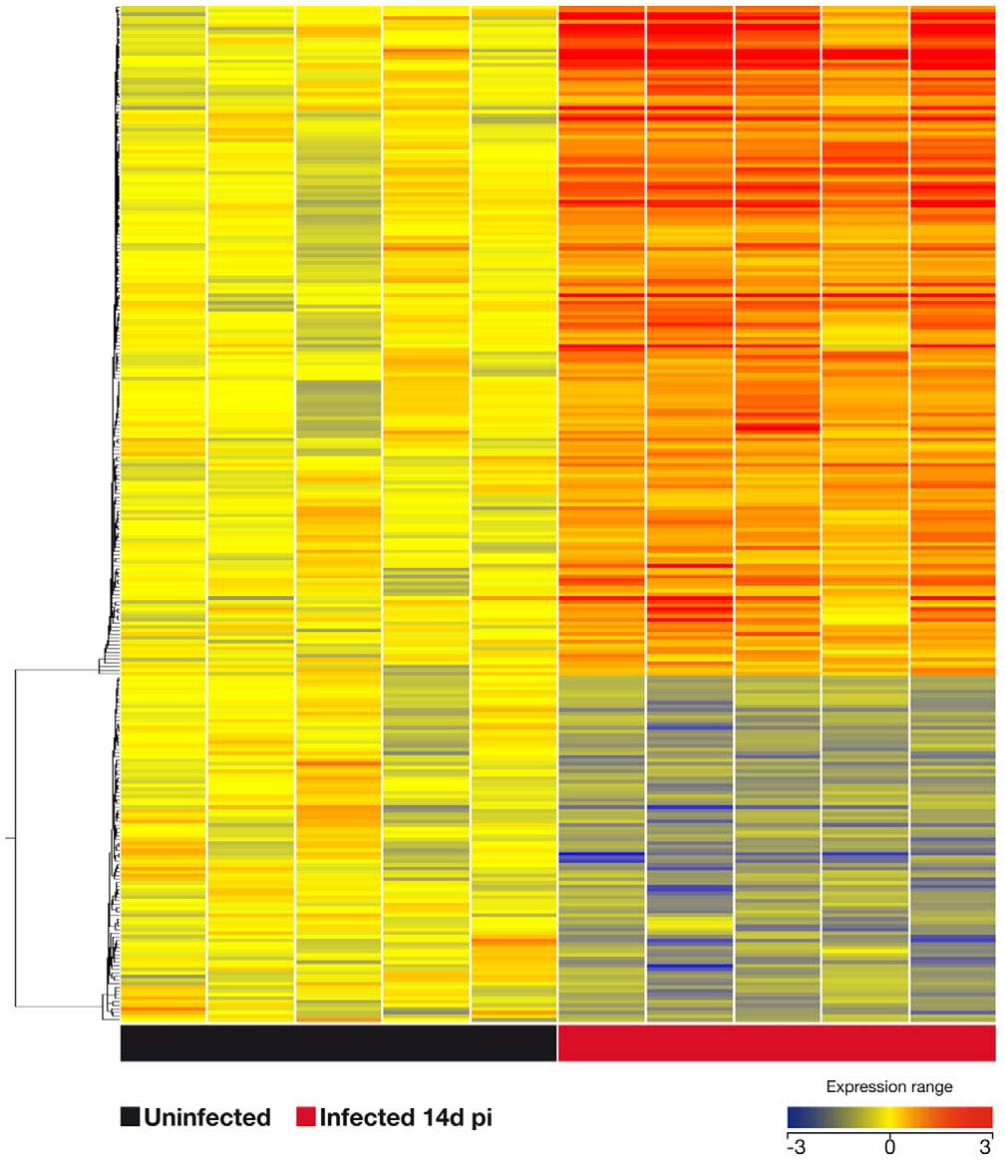
Pulmonary gene signature after 14 days after infection with *M. bovis*. The global transcriptional response in the lung of mice infected with *M. bovis* was compared the response of uninfected mice. Genes were considered significant when their correct p-value were below 0.05 with more than 2-fold change of expression. After 14 days post-infection the mice were euthanized and their lung cells were stimulated in vitro for 3 days with M7 protein pool (*see* *Materials and Methods*). Unsupervised hierarchical cluster was performed using a centroid linkage with a Person centered measure showing that 282 of these genes were modulated (see table S2 for list of these genes).

In antigen-stimulated lung cells we found 282 genes that were significantly modulated after 14 days post-infection (see Table S2 for list of genes and Figure 2 for heat-map of this signature of 282 genes). As expected [9], ifn-γ was strongly up-regulated (82-fold) in the lungs of infected animals after 14 days p.i. compared with naïve mice. Following the same trend were il-22 and cxcl9 with 74-fold and 22-fold change in their expression, respectively (Tables 1 and S2). Other genes that were differentially expressed in lungs after *M. bovis* infection were granzyme B, lymphocyte activation gene-3, il-17E receptor, and ccr6 (Tables 1 and S2).

### Pathway Analysis

**Figure 3:**
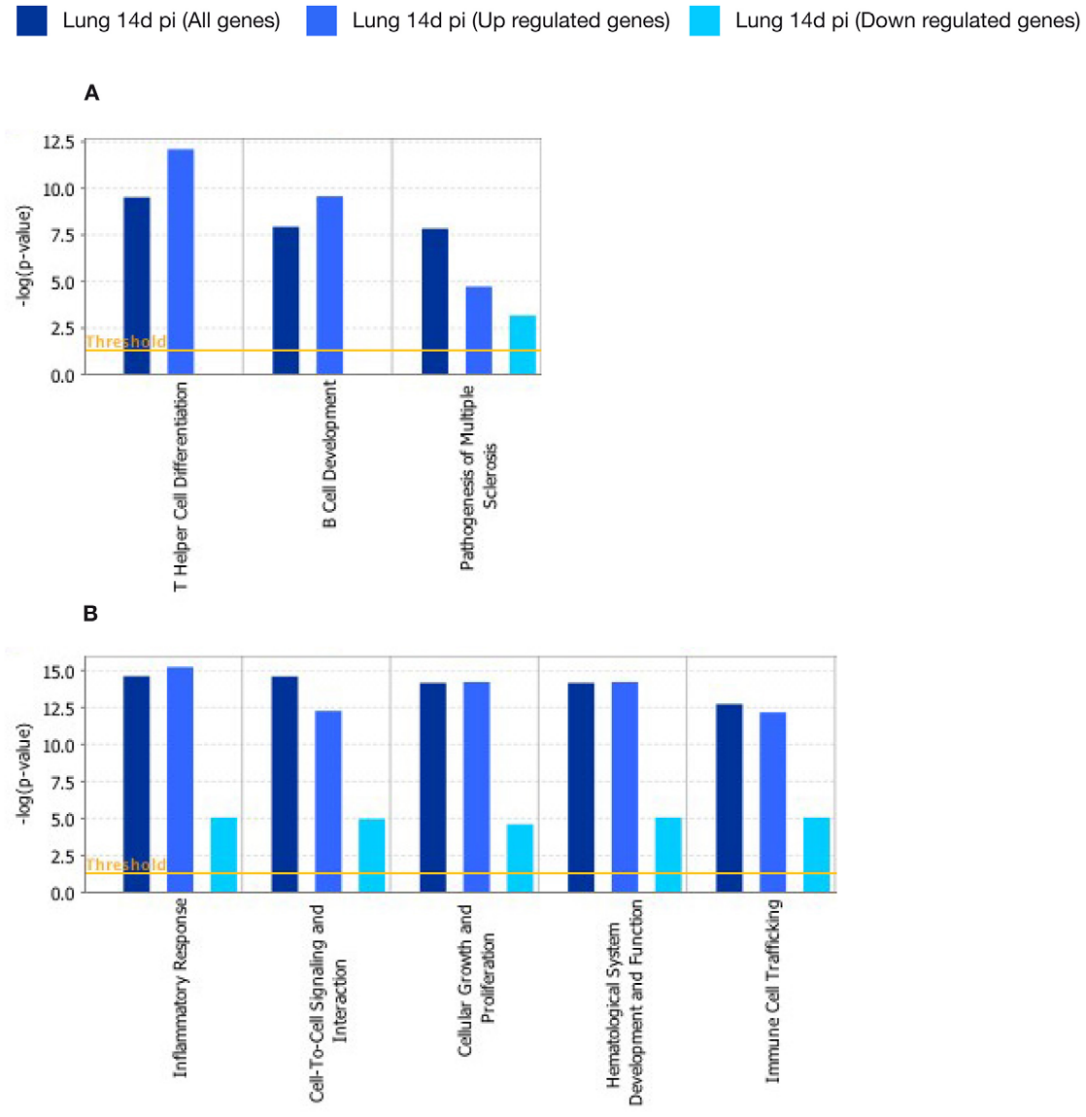
Functional networks (A) and canonical pathways (B) most significantly modulated in lung cells 14 days after *M. bovis* infection. Visualization of the trend and significance in the regulation of each network and pathway. Dark blue: all genes represented in a network. Light blue: genes that were up-regulated in a network. Cyan: gene those were down-regulated in a network. Fisher's exact test threshold value of p≤0.05.

Pathway Analysis using IPA was performed on the 282 genes that were significantly modulated in antigen-stimulated lung cells after 14 days post-infection. The two most significantly associated canonical pathways were related to T Helper Cell Differentiation (−log[p-value] = 9.47E00 and ratio = 1.53E−01) and B Cell Development (−log[p-value] = 7.88E00 and ratio = 1.53E−01) (Figure 3A). The five networks most significantly associated with these genes were inflammatory response (60 genes, p-value = 2.83E−15), Cell-To-Cell Signalling and Interaction (63 genes, ρ-value = 2.95E−15), Cellular Growth and Proliferation (77 genes, p-value = 8.2E−15) and Hematological System Development and Function (76 genes, p-value = 8.2E−15) (Figure 3B).

**Figure 4:**
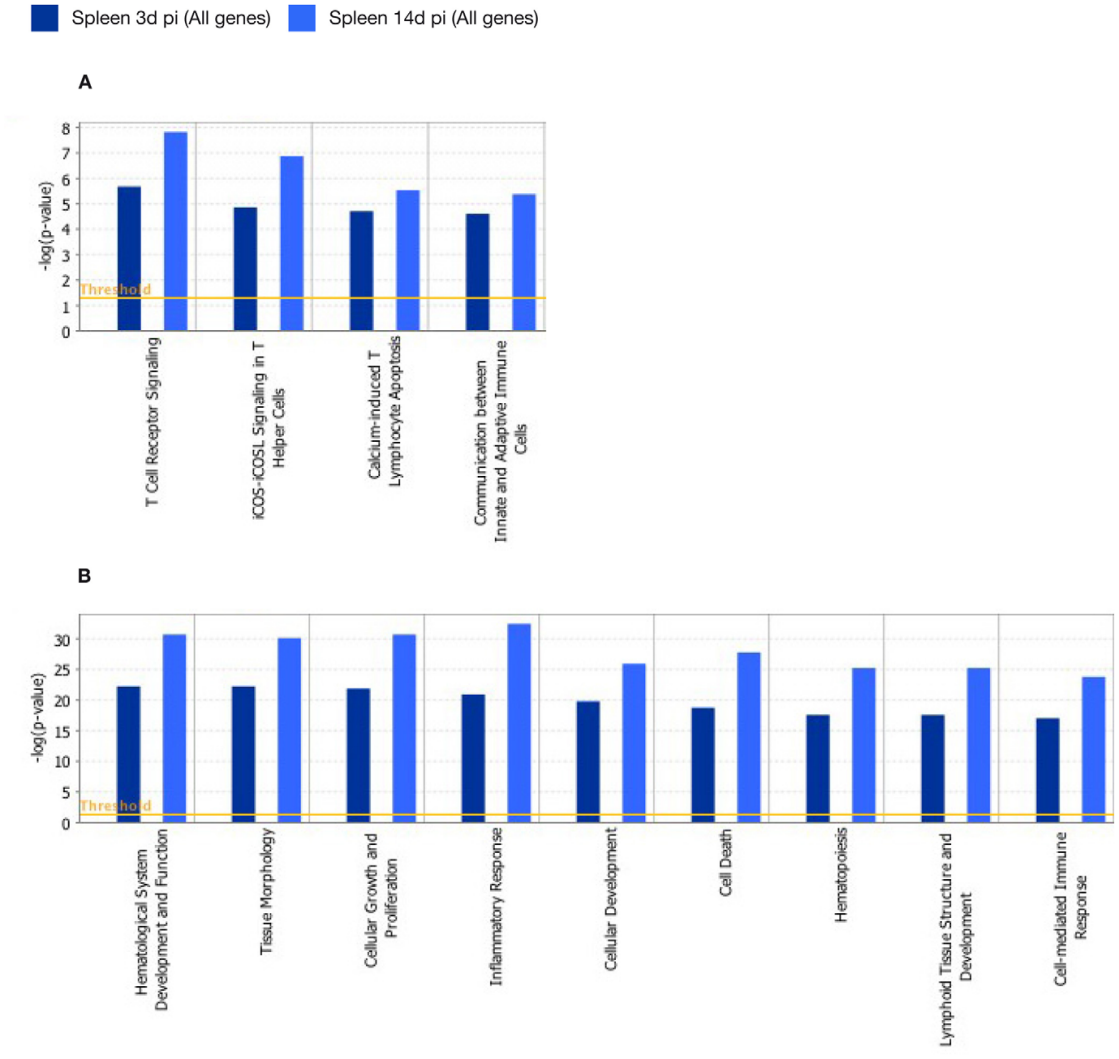
Functional networks (A) and canonical pathways (B) most significantly modulated in spleen cells 3 and 14 days after *M. bovis* infection. Visualization of the trend and significance in the regulation of each network and pathway are shown. Specific networks and pathways after 3 (dark blue bars) or 14 days p.i. (light blue bars) are indicated. Fisher's exact test threshold value of p≤0.05.

In antigen-stimulated splenocytes at 3 and 14 days p.i. we found statistically significant modulation of genes associated with the following dominant canonical pathways contained genes associated with T cell receptor Signalling (3 days p.i.: −log[p-value] = 5.65E+00 and ratio = 1.65E−01; 14 days p.i.: −log[p-value] = 7.8E00 and ratio = 1.93E−01) and iCOS-iCOSL Signaling in T Helper Cells (3 days p.i.: −log[p-value] = 4.83E00 and ratio = 1.39E−01; 14 days p.i.: −log[p-value] = 6.84E00 and ratio = 1.64E−01) (Figure 4A). Statistically significant modulation of genes of the following networks was also observed: Inflammatory Response (3 days p.i.: 175 genes, ρ-value = 7.51E−23; 14 days p.i.: 194 genes, p-value = 4.35E−33), Cellular Growth and Proliferation (3 days p.i.: 278 genes, p-value = 1.73E−22; 14 days p.i. : 289 genes, p-value = 2.51E−31), Hematological System Development and Function (3 days p.i.: 219 genes, p-value = 7.51E−23; 14 day p.i.: 235 genes, p-value = 2.51E−31), Tissue Morphology (3 days p.i. : 130 genes, p-value = 7.51E−23; 14 days p.i.: 142 genes, p-value = 1.59E−06), and Cell Death (3 days p.i.: 210 genes, p-value = 2.47E−19; 14 days p.i.: 227 genes, p-value = 2.09E−28) (Figure 4B).

**Figure 5:**
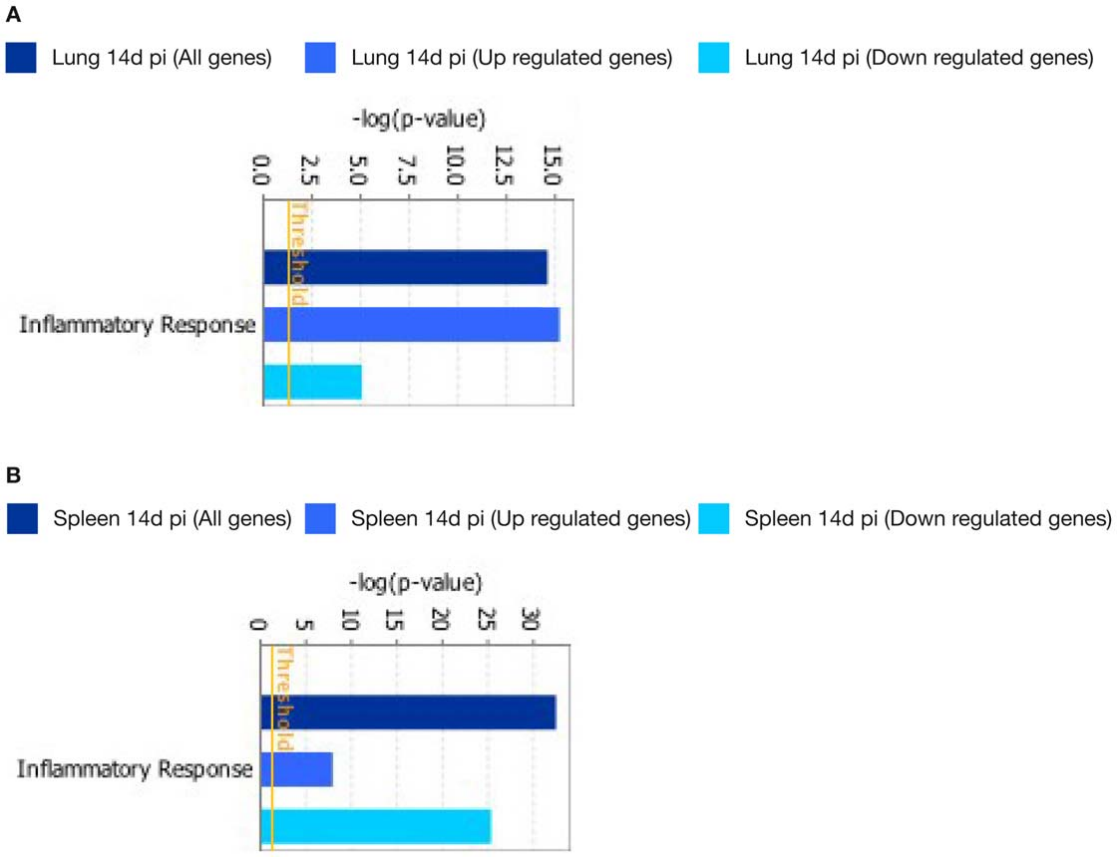
Induction of the inflammatory response network in lung (A) and spleen (B) cells at 14 days p.i. Dark blue: all genes enriched in this network. Light blue: genes that were up-regulated in this network. Cyan: gene those were down-regulated in this network.

Interestingly, when the networks and pathways associated with infection at the 14 days p.i. time point were compared between lung and spleen, the genes enriched in these pathways showed up-regulated expression in lung cells. In contrast, the same networks and pathways in spleen cells were enriched for genes whose expression was down-regulated. For example; whilst in the lungs the inflammatory response network is mainly represented by up-regulated genes, down-regulated genes dominate in the same network in spleen cells. Similarly, up-regulated genes were enriched in the T helper cell differentiation canonical pathway in lungs from infected mice, but in spleens the genes enriched in the same canonical pathway were predominantly regulated genes (Figure 5A and B).

### Validation of differential gene expression in natural infected cattle by qPCR

Our principal translational objective was the identification of biomarkers with potential application for blood-based ante-mortem diagnosis of bTb in cattle. Based on our previous results [10], [11], we hypothesized that results obtained in our mouse model could guide the selection of such markers for cattle. Thus, we evaluated the expression of a selection of genes most strongly up-regulated in the mouse experiments described above (Table 1) in cattle using PBMC from uninfected and cattle naturally infected with *M. bovis*. Apart from genes expressed in the lung of infected mice (14 days p.i.), we also selected genes from the murine spleen that were up-regulated both early and late after infection (3 and 14 days p.i.). IL17A was included as positive control as it had been shown previously to be associated with infected cattle [10], [12], [13]. RNA was prepared from PBMC cultures stimulated with PPD-B and the expression of these genes evaluated by qRT-PCR. The results are shown in Table 1. Five of the 14 genes selected based on the murine transcriptome analyses described above were found to be also significantly up-regulated in bovine PBMC from infected animals compared to naïve controls. The most highly modulated genes were those encoding IFN-γ, IL22, CXCL9, CXCL10, and GzmA (Table 1). The other 9 genes studied were not significantly modulated in bovine PBMC from infected animals (Table 1). The gene encoding for IL17A was also expressed stronger in bTb infected cattle compared to TB-free cows, although its expression was not quite statistically significant (P = 0.0052, Table 1).

When we compared the expression of the genes encoding IFN-γ, IL22, CXCL9, CXCL10, GzmA, and IL17A, we did not find correlations between their expression the disease severity described by the pathology scores [14] assigned after post *mortem* examinations of the infected cattle (data not shown). However, this study was not designed primarily to correlate expression levels with disease severity and we therefore acknowledge that its statistical power was not sufficient to avoid type 2 errors. Larger animal numbers, including experimentally infected cattle need to be tested to validate this hypothesis in greater details.

**Figure 6:**
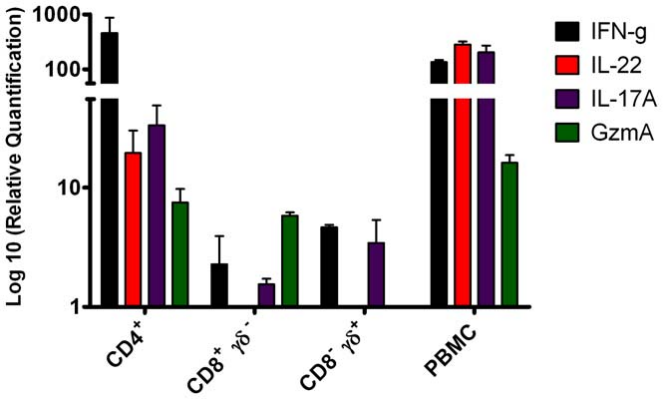
Phenotypic analysis of bovine T cell subsets that expressed the genes encoding for IL22, IL17a and GzmA. Highly purified CD4^+^, CD8^+^, and CD8^−^/TCRδ^+^ T cell populations were isolated by FACS sorting and the mRNA expression of these genes was determined after stimulation with PPD-B. Results are presented as mean fold increase compared to media control values ± SEM.

In a final set of experiments we determined the phenotype of the bovine T cell subset(s) that transcribed the genes for IL22, IL17A and GzmA. IFN-γ in this system is exclusively produced by CD4^+^ T cells (Vordermeier, unpublished data) and was used as control. Highly enriched CD4^+^, CD8^+^ and TCRγδ^+^ (γδ^+^) T cell subset populations were isolated by FACS sorting and co-cultured in the presence of CD14^+^ monocytes were as APC and PPD-B. The expression of these genes was determined 24 hours later by qRT-PCR. The results (Fig. 6) demonstrated that ifn-γ and il22 were expressed by bovine CD4^+^ T cells. However, whilst il17A was also predominantly expressed by CD4^+^ cells; CD8^+^ and γδ^+^ T cells also expressed some il17A albeit an order of magnitude less. Granzyme A expression could be detected in both CD4^+^ and CD8^+^ T cell subsets (Figure 6).

## Discussion

BTb remains an economically import disease of livestock species and improved diagnostic tests would benefit the implementation of control strategies. Transcriptomics approaches have been used to identify gene expression profiles to define biomarkers of TB in mice, primates and humans in different infection conditions. Several recent publications have reviewed these studies [15], [16]. Likewise, studies in cattle, aiming to determine gene expression profiling, have been reviewed by Waters *et. al.* [17] focusing on *ex vivo studies and* macrophage infection. Yet, the interaction between host and *M. bovis*, which result in bTb, remains poorly characterized in cattle. The definition of such biomarkers induced after infection could have impact on the rational design of novel diagnostic approaches. We have used the advantages of the murine model (cheap, relatively short experimental periods, availability of reagents, detailed genome annotation) to study the host transcriptome after *M. bovis* infection. As we hypothesised our analysis has lead to the validation in cattle of a number of biomarkers found in the murine system which include the genes encoding IL22, IL17A and Gzm A. PPD-B was used to stimulate bovine PBMC because it is the standard antigen used to diagnose bTb in livestock. In contrast, a protein cocktail (M7) was used to stimulate mouse lymphocytes because PPD-B is a poor antigen to stimulate murine responses despite the presence of these proteins in PPD-B (Hogarth *et al.*, unpublished observation). The reasons underlying this discrepancy in antigenic activity between the two species are not clear at present. Furthermore, the objective was to define bovine biomarkers applicable to routine ante-mortem blood based bTb diagnosis in cattle. Therefore, we targeted our analysis to peripheral blood as the only practical sample that can be collected readily from cattle in the field.

Interestingly, we could not validate in cattle the over-expression of all genes that we prioritised based on the mouse experiments. This could be due to the fact that we studied peripheral blood responses in cattle, whilst the mouse studies concentrated on tissues (lung and spleen). In addition, the infection status is likely different between the two species populations studied: Murine responses were assessed relatively early after infection, whilst the time of infection in the cattle studied cannot be defined as these animals were naturally infected and are likely composed of a very heterogeneous group. It is therefore possible that not all responses found at the tissue sites of infection are reflected in the blood. However, our results demonstrated the value of the mouse system to guide the study of gene expression in cattle.

Our data also suggested that the networks and pathways associated both in lungs and spleen with infection at the 14 days p.i. time point showed up-regulated expression in the lung whilst the same networks and pathways in spleen cells were enriched for genes whose expression was down-regulated. This could be explained by sequestration of particular cell populations into the lungs as principal site of infection as we previously proposed [18]. As our principal objective was to define bovine biomarkers useful for the ante-mortem diagnosis of bTb in cattle, we concentrated on the validation of potential markers that were strongly up-regulated in spleen and lung cells to allow the assessment of the widest selection of markers possible. In addition to their over-expression in mice, we also selected genes for validation in the bovine system on those whose products would have the potential to be detected by antibody-based assay systems such as ELISA. This lead therefore to the prioritisation of markers such as chemokines and cytokines.

IL-22 belongs to the IL-10 cytokine family and is produced by NK cells, mast cells, and T cells, especially Th17 and Th22 cells. It is involved in the antimicrobial defence of mucosal surfaces including in the airway by promoting innate immunity to bacterial infection. IL-22 has been shown to induce genes encoding antimicrobial proteins, β-defensins, S100 calcium binding proteins and to up-regulate the expression of chemokine (ccl1, cxcl5 and cxcl9) and cytokine genes (il-6 and g-csf). Furthermore, the functional consequences of IL-22/IL22R signalling can be potentiated by IL-17A/F and TNF-α in order to promote the expression of many of the genes encoding molecules involved in host defence in the lung [19]. The specific role of IL-22 in *M. tuberculosis* infection remains undefined, although it has been reported that IL-22 did not have a significant role in host protection and granuloma formation in mice [20], [21]. In contrast, the production of IL-22 by human NK cells or addition of recombinant IL-22 to infected macrophages enhanced phagolysosomal fusion and reduces growth of *M. tuberculosis* [22]. In addition, CD4^+^ T effector cells bearing membrane-bound IL-22 (IL22^+^ CD4^+^ T cells) significantly reduced intracellular *M. tuberculosis* replication in macrophages isolated from rhesus macaques [23]. Whether IL-22 contributes to control TB at the site of infection is unknown. Nevertheless, active TB is associated with lower frequencies of IL17^+^ and IL22^+^ CD4^+^ T cells in peripheral blood [24]. In contrast, we have also demonstrated elevated expression of IL22 in lymph nodes from infected cattle (Aranday-Cortes et al., unpublished).

The role of IL17A in human tuberculosis has been described (reviewed in [25], [26]. Although IL-17 might not play an equally important role in protection against mycobacterial infections as IFN-γ [27], recent studies have shown that IL-17 mediates an inflammatory response through granulopoiesis and consequent neutrophil accumulation. This may be required for protective immunity in the early stage of infection but could also become detrimental if its production remains high during later stages of disease [28]. In addition, IL-17 is reported to play an essential role in the formation of granulomas in mice infected with BCG [29] and reduced Th17 CD4 T cell numbers are associated with PPD-induced impaired cytokine response in patients with HIV [30]. IL-17 producing γδ T cells were increased in patients with active pulmonary tuberculosis [31]. Interestingly, we have recently described a role for IL17A in the protective immune responses against bTb in cattle after BCG and BCG/viral subunit prime-boost experiments both in cattle [10] and mice [11]. In cattle its expression was up-regulated in protected animals after vaccination but before challenge and can therefore be defined as predictor of protection [10]. In this study we now also define it as a marker of disease progression in cattle and mice. Our data therefore are in agreement with an earlier report by Blanco et al. [13] who reported that il-17 expression was positively associated with pathology in cattle. Thus, our observations also support the notion that IL-17A is involved both in immune processes leading to protection or immunopathology of tuberculosis.

Granzyme A is the most abundant of the cytotoxic granules released by cytotoxic T cells (CTL) and NK cells. It can induce cell death independently of caspase activation [32], [33] and T cells can reduce intracellular growth of *M. tuberculosis* by perforin and Fas/Fas ligand independent mechanism even in IFN-γ and TNF-α deficient mouse models [34], [35]. One hypothesis which explains these observations, is that Th17/Th22 T cells may play a role in the pulmonary inflammatory response post-infection, by helping to elicit a pathogenic inflammatory response involving the activation of CTL. A similar relationship between TH17 cells and CD8^+^ CTL has been described in tumour immunology [36].

Whilst it is acknowledged that IL-17A and IL-22 are produced not only by CD4^+^ T cells, but also by CD8^+^ and γδ T cells (as well as NK, NKT and non-T cells, which were not evaluated in this study) [37], [38], [39], [40], [41], our data demonstrated that both cytokines were predominantly expressed by CD4^+^ T cells with a minor il-17A response also attributable to γδ T cells. It is therefore tempting to speculate that the responding bovine CD4^+^ T cells belong to Th17 and/or Th22 subsets comparable to those described in other species [42], [43]. Granzyme A was expressed equally in CD4^+^ and CD8^+^ which is suggestive of the induction of CTL of both T cell subsets following bTb infection of cattle.

Comparison between spleen samples obtained at 3 and 14 days p.i. and lungs samples at 14 days p.i. demonstrated that 32 genes were significantly modulated in all three sets of samples (Tables S1 and S2, genes highlighted with asterisk). Interestingly, only 4/32 genes were up-regulated (ubiquitin, lymphocyte antigen 6 complex, fmrd4 and ccr5). CCR5 is a chemokine receptor that binds CCL3, CCL4 and CCL5. The interaction between CCR5 and CCL5 may play an early protective role in limiting *M. tuberculosis* growth by recruiting T-cells, NK and macrophages to the lungs [44]. Further, reduction in number and frequency of Th1/Th17 CCR5^+^ T cells was associated with reduced IFN-γ and IL-2 PPD responses in HIV-infected patients [30]. In contrast, severe TB in juvenile rhesus monkeys was associated with the up-regulation of ccr5 (as well as il-22 and other inflammatory cytokines and receptors) [45]. Thus, its precise role in tuberculosis is therefore unclear to-date.

Chemokines such as CXCL10 have been used as additional read-outs for blood-based IFN-γ release assays of human tuberculosis [46], [47]. Its application increased overall test sensitivity compared to IFN-γ alone [48]. Measuring cxcl9 and cxcl10 expression by qRT-PCR has also been reported as potential platform to increase diagnostic sensitivity in human tuberculosis [49]. It is therefore interesting that we could show up-regulation of both of these genes also in murine lung cells and in bovine PBMC. Further validation of our results in cattle will determine whether these, or the other genes validated in cattle such as il-22 or their protein products, will increase the accuracy of blood-based diagnostic tests for bTb when applied alongside IFN-γ release assays. However, the biomarkers identified in our study have so far been prioritised only based on their increased gene expression levels. Confirmation of their increased production at the protein level will be needed to turn them into valid diagnostic tests for bTb. Development of such antibody-based detection systems is now part of the process of translating our findings into practical such as ELISA-based, diagnostic tests for bTb.

In conclusions, we have shown that biomarkers defined in the murine system can be used to guide the analysis of biomarkers of disease in cattle. Further, we have prioritised a number of cytokines and chemokines as potential additional diagnostic markers for the blood based ante-mortem diagnosis of bTb to improve traditional IFN-γ release assays.

## Materials and Methods

### Ethics

This study and all procedures were approved by the Animal Health and Veterinary Laboratories Agency (AHVLA) Animal Use Ethics Committee (UK Home Office PCD 70/6905) and performed under appropriate personal and project licences within the conditions of the Animals (Scientific Procedures) Act 1986. All animals were housed in appropriate biological containment facilities at the AHVLA.

### Animals

#### Mice

Female BALB/C mice were obtained from SPF facilities at Charles River Laboratories, Margate, UK.

#### Cattle

Heparinized blood samples were obtained from 11 naturally infected single intradermal comparative tuberculin test (SICTT)-positive reactors from herds know to have bTb. Infection was confirmed by the presence of visible pathology at post-mortem and the culture of *M. bovis* from tissues from these animals according to previously described procedures [14]. *Uninfected controls*: Heparinized blood sample were obtain from 9 SICTT-negative animals from bTb-free herds. They were also negative in the standard Bovigam IFN-γ release assay (Prionics, Switzerland).

### Antigens

#### Cattle

Purified protein derivative from *M. bovis* (PPD-B, Prionics, Switzerland) was used in culture at 10 µg/ml for stimulating bovine Peripheral blood mononuclear cells (PBMC). Staphylococcal enterotoxin B (SEB, Sigma-Aldrich, UK) was used as a positive control at 1 µg/ml.

#### Mice

Antigen cell culture stimulations in mice were performed using an equal pool of seven secreted, immunogenic recombinant mycobacterial proteins (Rv1886c, Rv3019c, Rv3763, Rv3804c, [Lionex GmbH, Germany] Rv0251, Rv0287 and Rv0288 [Proteix s.r.o., Czech Republic]) common to *M. bovis* and BCG, referred here as M7 protein cocktail. We have previously shown that M7 induced strong and representative T cell responses in both vaccinated and infected mice [18]. Each protein was used at final concentration of 2 µg/ml in 3-day culture. Concanavalin A (Sigma-Aldrich) was used as a positive control at 5 µg/ml for murine cells.

### Mycobacterial challenge

*Mycobacterium bovis* isolate AF2122/97 was grown to mid log phase in Middlebrook 7H9 broth supplemented with 4.16 g/L pyruvic acid, 10% (v/v) oleic acid, albumin, dextrose, and catalase (OADC) and 0.05% (v/v) Tween 80, subsequently stored at −80°C, was used for all virulent challenges. Two groups of 5 mice each were challenged with approx 600 CFU via the intranasal route [50]. At days 3 and 14 post challenge five mice per group were euthanized and spleens and lungs harvested aseptically.

### Cell isolation

Spleen and lung cells were prepared by as described previously [18] and suspended at 5×10^6^/ml spleen cells and 5×10^5^/ml lung cells. After stimulation, cells were washed (300× *g*, 5 min at room temperature) and supernatants removed. One ml of Trizol (Invitrogen, Paisley, UK) was then added and the cell lysates were stored at −80°C.

#### Bovine PBMC

PBMC were isolated from heparinized blood by Histopaque-1077 (Sigma-Aldrich) gradient centrifugation. Cells were resuspended at 1×10^6^/ml in tissue culture medium (RPMI 1640 [Sigma-Aldrich] supplemented with 10% fetal calf serum [Sigma-Aldrich], nonessential amino acids [Sigma-Aldrich], 100 U/ml penicillin and 100 µg/ml streptomycin sulfate) and incubated overnight with PPD-B or SEB in 24-well tissue culture plates (Life Technologies, UK). The following day, plates were centrifuged (300× *g*, 5 min at room temperature) and the supernatant was removed. One ml of Trizol was then added and the cell lysates were stored at −80°C.

### RNA extraction from bovine PBMC

Total RNA was extracted from PBMCs using TRIzol according to the protocol recommended by the manufacturer. Turbo DNA-free (Ambion, Huntingdon, UK) was used to remove genomic DNA contamination. The purity and concentration of RNA were evaluated by NanoDrop 1000 (Thermo Scientific, Horsham, UK). RNA with a ratio of A260/A280 ≥1.7 was used for the RT-qPCR validation study.

### Cell Sorting

Cell sorting was performed using a Beckman Coulter MoFlo Astrios instrument. Bovine PBMCs were stained and sorted according to expression of the bovine T-cell surface markers CD4, CD8 and γδ TCR. The anti-bovine CD8 antibody (clone CC63) was supplied directly conjugated to Fluorescein isothiocyanate (AbD-Serotec, Kidlington, UK), whilst the anti-bovine CD4 antibody (clone CC8, AbD-Serotec) was custom conjugated to R-Phycoerythrin (Invitrogen). The anti-TcR-δ antibody (clone GB21A, VMRD, Pullman, WA) was used in the primary staining reaction as an unconjugated antibody and then labelled in a secondary staining reaction using an isotype specific anti-mouse IgG2b antibody directly conjugated to Alexa Fluor 633 (Invitrogen). Staining reactions were performed at 4°C for 15 minutes. CD14-positive cells were isolated using magnetic beads (Miltenyi Biotech, Bisley, UK) as described previously [51] and used as antigen-presenting cells. The purities of the sorted T cell populations were >99.% for CD4^+^CD8^−^ cells; >96% for TcR-δ^+^ CD8^−^ cells and >99.0% for TcR-δ^−^ CD8^+^ cells. In the assays, 1×10^6^ sorted T cell populations were incubated for 24 h with 1×10^5^ APC in 24-well plates in the presence of PPD-B and RNA processed as described above.

### Murine RNA preparation and microarray hybridization

Spleen and lung cells were collected into Trizol and stored at −80°C until further processing. RNA was isolated from spleen and lung cells derived from control and infected mice using standard RNA extraction protocols (Miltenyi Biotech). The quality of RNA samples was assessed using the Agilent 2100 Bioanalyzer platform (Agilent Technologies, UK). All RNA samples revealed acceptable RNA Integrity Number (RIN) values of between 7.4 and 9.6. For the linear T7-based amplification step, 0.06 µg–0.5 µg of each total RNA samples was used as starting material. To produce Cy3-labeled cDNA, RNA samples were amplified and labeled using the Agilent Low RNA Input Linear Amp Kit (Agilent Technologies) following the manufacturer's protocol. Yields of cRNA and the dye-incorporation rate were measured in a ND-1000 Spectrophotometer (Thermo Scientific). In general, control samples were labeled with Cy3 and experimental samples were labeled with Cy5. The hybridization procedure was performed according to the Agilent 60-mer oligo microarray processing protocol using the Agilent Gene Expression Hybridization Kit. Briefly, 825 ng Cy3- and Cy5-labeled fragmented cDNA in hybridization buffer was hybridized overnight (17 hours, 65°C) to Agilent Whole Mouse Genome Oligo Microarrays 4x44K using Agilent's recommended hybridization chamber and oven. Finally, microarrays were washed once with 6× SSPE buffer (3.6 M NaCl, 0.2 M NaH_2_PO_4_, 0.02 M EDTA pH 7.4) contaning 0.005% N-lauroylsarcosine for 1 min at room termperature followed by a second wash with preheated 0.06× SSPE buffer (37°C) containing 0.005% N-lauroylsarcosine for 1 min. The last washing step was performed with acetonitrile for 30 seconds.

### Normalization, filtering procedures and data analysis

Fluorescence signals of the hybridized Agilent Microarrays were detected using Agilent's Microarray Scanner System. Agilent's Feature Software (FES) was used to read out and process microarray image files. The software determines feature intensities (including background subtraction), rejects outliers and calculates statistical confidences. For determination of differential gene expression FES derived output data files were further analyzed using GeneSpring GX 11.5 (Agilent Technologies). After baseline transformation to mean of control samples (spleen and lung from uninfected mice), we decided to focus on those genes that reliably change their expression, then we filtered the microarrays following three conditions: 1) *Filter by value*. Genes that do not have normalized signal intensity values of more than −0.5 and 0.5 were disregarded. 2) *Filter by flags*. All the genes with flags values present in at least 100% of the values in any 1 out of the 3 conditions were considered. 3) Filter by percentile. All the genes with raw signal intensity values between 25 and 100 in any 1 out of the 3 conditions were also considered. Finally, all the genes in coincidence between filtering by flags group and filtering by percentile group were kept for statistical analysis.

After filtering, parametric analysis of variance was applied to compare mean expression levels in each analysis. Data were considered significant when the Benjamini Hochberg false discovery rate (FDR) for the comparison under analysis was <0.05, and the significance level was <0.05. In order to focus on highly regulated genes, we also restricted the majority of the analysis to genes with changes in expression levels of at least 2.0-fold change (FC) in all the conditions. All data set can be downloaded from Gene Expression Omnibus public data base at www.ncbi.nml.nih.gov/geo/ with the GEO accession number GSE33058.

Lists of genes resulting from these analyses were submitted to Ingenuity Pathway Analysis (IPA; Ingenuity® Systems, USA, www.ingenuity.com). In order to identify the most significant functional networks (biological functions and diseases) and canonical pathways related to each comparison, the analysis was performed using the following strategy: A core analysis was performed with all the genes with ρ≤0.05 and fold change ≥2 for each comparison; then for the same comparisons a core analysis was performed only considering those genes that showed p≤0.05 and were at least 2 fold up-regulated. Further and independently a last core analysis was performed for those genes with p≤0.05 that were at least 2-fold down-regulated. Finally, these analyses were compared. Fisher's exact test with a threshold value of p≤0.05 was used in all the analyses. The rationale behind this strategy is to visualize the trend and significance in the regulation for each network and pathway. Thus, we show three columns for each comparison: one showing all the genes related to a specific network and pathway, followed by two extra columns showing how the networks and pathways are enriched by up- or down-regulated genes.

### Reverse transcriptase and quantitative Real-time PCR validation

cDNA from PBMCs was synthesized from total RNA samples using random primers and reverse transcription with SuperScript III Vilo reverse transcriptase following the manufacturers protocol (Applied Biosystem, Paisley, UK). cDNA from cell sorting was synthesized using μMACS One-step cDNA Kit (Miltenyi Biotec) following manufacturers instructions. Transcripts were quantified by qPCR with Fast SYBR Green master mix (Applied Biosystem) following manufactures conditions. qPCR analysis was performed using the ABI 7500 Fast Real Time PCR System (Applied Biosystem) in triplicate from media control, PPD-B and SEB-stimulated PBMCs cDNA. The fold increase was calculated by comparison with the expression of endogenous controls genes SDHA and G3PDH using the 2^−ΔΔct^ calculation [52].

### Statistical analysis

Responses between cattle naturally infected with *M. bovis* and naïve controls were analysed by Student's t-test on log transformed data using Prism (Graph Pad, USA). To control for type I errors due to multiple comparison, the Bonferroni's correction for multiple tests was applied and the significance level set at p<0.003.

## Supporting Information

Table S1 **Significant modulation of spleen cell genes early after 3 days p.i. (1109) and at later time-point of 14 days p.i. (1134).** Genes in bold (618) were modulated at both time points. Ns = no significant expression at this time point. The genes marked with (*) result common after comparison between spleen samples after 3 and 14 days p.i. and lungs samples after 14 days p.i. with M. bovis showed expression of (32).
(XLS)

Table S2 **Significant modulation of spleen cell genes early after 3 days p.i. (1109) and at later time-point of 14 days p.i. (1134).** Genes in bold (618) were modulated at both time points. Ns = no significant expression at this time point. The genes marked with (*) result common after comparison between spleen samples after 3 and 14 days p.i. and lungs samples after 14 days p.i. with M. bovis showed expression of (32).
(XLS)
